# Tanshinone IIA attenuates cognitive impairment in vascular dementia rats by modulating copper homeostasis via the SLC31A1/FDX1 axis

**DOI:** 10.1371/journal.pone.0356885

**Published:** 2026-08-24

**Authors:** Hongxiao Lv, Xinyu Yang, Yutong Liu, Junwei Zhou, Wentao Yu

**Affiliations:** 1 Hebei University of Chinese Medicine, Shijiazhuang, China; 2 Beijing Hospital of Traditional Chinese Medicine, Capital Medical University, Beijing, China; 3 Hebei International Joint Research Center for Dominant Diseases in Chinese Medicine and Acupuncture, Shijiazhuang, China; 4 Hebei Key Laboratory of Chinese Medicine Research on Cardio-Cerebrovascular Diseases, Shijiazhuang, China; Scuola Superiore Sant'Anna, ITALY

## Abstract

**Background:**

Tanshinone IIA (TSA) is the core liposoluble active component of *Salvia miltiorrhiza*, a traditional Chinese medicine, and exhibits multiple pharmacological activities including anti-inflammation, anti-oxidation, anti-apoptosis and mitochondrial function improvement. Studies have confirmed that TSA can ameliorate cognitive function in rats with vascular dementia (VaD) by alleviating cerebral ischemic injury, inhibiting neuroinflammation, protecting the blood-brain barrier and other pathways. However, it has not been reported whether TSA exerts its neuroprotective effect by regulating the cuproptosis pathway. Focusing on cuproptosis, this study investigated the effects of TSA on cognitive impairment, neuronal injury and cuproptosis-related mechanisms in a rat model of VaD.

**Methods:**

A rat model of VaD was established by permanent bilateral common carotid artery ligation (2-VO), and the model rats were treated with TSA. The cognitive impairment of VaD rats was evaluated by the Morris water maze test. Hematoxylin-eosin (HE) staining and Nissl staining were used to detect neuronal injury and loss in the hippocampus of VaD rats. Transmission electron microscopy (TEM) was performed to observe the ultrastructure of mitochondria in the hippocampus; Western blot (WB) assay was adopted to detect the expression levels of cuproptosis-related proteins, including solute carrier family 31 member 1 (SLC31A1), ferredoxin 1 (FDX1), lipoic acid synthetase (LIAS) and dihydrolipoamide transacetylase (DLAT).In vitro, an oxygen-glucose deprivation/reoxygenation (OGD/R) model was established in HT-22 cells to evaluate the effects of TSA on cell viability (CCK-8), intracellular reactive oxygen species (ROS) levels, and copper accumulation (Coppersensor-1 staining), further validating the involvement of copper homeostasis in TSA-mediated neuroprotection.

**Results:**

The results revealed that TSA markedly alleviated cognitive dysfunction and neuronal damage in VaD rats. It also reversed the cuproptosis-related abnormal changes in the brain of VaD rats, and modulated the expression of SLC31A1/FDX1 pathway proteins. In vitro, TSA protected HT-22 cells from OGD/R-induced injury by reducing oxidative stress and copper accumulation.These changes were associated with amelioration of copper overload and mitochondrial damage, suggesting that TSA may exert neuroprotection partially through the regulation of cuproptosis-related pathways.

**Conclusion:**

TSA effectively alleviated cognitive impairment and neuronal injury in VaD rats, and exerted neuroprotective effects both in vivo and in vitro by attenuating copper-dependent cytotoxic stress, preserving mitochondrial integrity, and modulating the SLC31A1/FDX1 axis. This study provides a new perspective and theoretical basis for the future development of targeted drugs for VaD.

## 1. Introduction

Vascular dementia (VaD) is a cognitive impairment syndrome caused by cerebrovascular diseases or vascular risk factors. It is the second most common type of dementia worldwide after Alzheimer’s disease, accounting for approximately 15%–20% of all dementia cases [1]. Its core pathological basis is neuronal loss and brain function degeneration induced by chronic cerebral hypoperfusion and cerebrovascular injury [2]. The clinical manifestations include memory decline, inattention, executive dysfunction, etc., which seriously affect the quality of life of patients and impose a heavy medical burden on families and society [3]. Although some understanding of the pathological mechanisms of VaD has been achieved, there are still no specific therapeutic drugs. Clinical interventions are mostly limited to controlling risk factors and alleviating symptoms, which cannot effectively delay disease progression. Therefore, further revealing its novel pathological mechanisms and developing targeted therapeutic strategies on this basis is of great clinical significance and scientific value for improving the prognosis of patients.

VaD is pathologically heterogeneous, encompassing multiple subtypes including subcortical ischemic vascular dementia (SIVD), multi-infarct dementia, and strategic single-infarct dementia [4]. Neuropathologically, VaD is characterized by multifocal and/or diffuse lesions, ranging from lacunar infarcts and microinfarcts to diffuse white matter changes involving myelin loss and axonal abnormalities, often affecting subcortical structures, basal ganglia, thalamus, and white matter tracts [5,6]. Among these, chronic cerebral hypoperfusion (CCH) is recognized as a primary driver of vascular cognitive impairment, with cerebral blood flow typically falling below 24–45 mL/100 g/min (normal: 50–60 mL/100 g/min) [7].

To investigate the pathophysiology of VaD and evaluate potential therapeutic interventions, several animal models of chronic cerebral hypoperfusion have been developed. Among these, the permanent bilateral common carotid artery occlusion (2-VO) model in rats is one of the most widely used and well-characterized approaches [8,9]. The 2-VO model exhibits characteristic features of subcortical ischemic VaD, including white matter damage, neuronal shrinkage in the cerebral cortex and hippocampus, and cognitive impairments in spatial learning and memory tasks [10]. Importantly, the 2-VO rat model has been validated as a useful tool for investigating the pathophysiology of human dementia and for elucidating the therapeutic potential of candidate drugs [9]. However, this model also possesses inherent limitations: cerebral blood flow drops sharply and substantially after acute ligation of the common carotid arteries, and the model lacks concomitant vascular risk factors (e.g., hypertension, diabetes) and causative small vessel changes that typically characterize human VaD [11]. Despite these limitations, the 2-VO model remains a standard and reproducible approach for initial mechanistic screening of neuroprotective agents in VaD, given its well-defined timeline of pathological progression, established cognitive endpoints, and extensive historical data for cross-study comparison [8,9].

Dysregulated cell death modalities play a pivotal role in the pathological progression of VaD. In recent years, cuproptosis has been identified as a novel regulated form of cell death, characterized by mitochondrial dysfunction, abnormal aggregation of lipoylated proteins and loss of iron-sulfur cluster proteins triggered by aberrant copper ion accumulation, ultimately leading to proteotoxic stress and cell death [12]. As an essential trace element in the organism, copper homeostasis relies on the precise regulation of transporters. Among these, SLC31A1 mediates the intracellular influx of copper ions. FDX1, as a core regulatory factor of the cuproptosis pathway, not only reduces Cu²⁺ to the more toxic Cu ⁺ , but also modulates the post-translational modification of downstream lipoylated proteins (e.g., dihydrolipoamide transacetylase, DLAT), thereby directly driving the onset and progression of cuproptosis [13]. Recent studies have indicated a close association between the cuproptotic mechanism and neurological disorders [14]. In VaD models, cerebral vascular injury can induce copper homeostasis imbalance, exacerbate neuronal damage by activating the cuproptosis pathway, and inhibition of cuproptosis is promising to ameliorate neurological deficits, suggesting that cuproptosis may serve as a potential therapeutic target for VaD. Furthermore, the SLC31A1/FDX1 signaling pathway, as the core axis regulating cuproptosis, has been proven to be abnormally activated and involved in the pathological processes of various diseases [15]. Downregulation of this pathway can effectively inhibit copper ion accumulation and the transduction of downstream death signals, providing a well-defined molecular target for cuproptosis-targeted therapy [16].

TSA is the major liposoluble active component of Salvia miltiorrhiza, a traditional Chinese medicine. It exhibits prominent anti-inflammatory, antioxidant, mitochondrial protective and neuroprotective effects, and has been widely used in the basic research and clinical treatment of cardio-cerebrovascular diseases [17]. Studies have confirmed that TSA can significantly alleviate cognitive impairment in animal models of VaD through mechanisms such as improving cerebral perfusion, inhibiting oxidative stress and reducing neuronal apoptosis [18]. However, it remains unclear whether its protective effects are associated with the regulation of cuproptosis. Considering the potential of TSA in regulating intracellular homeostasis and the pathological role of cuproptosis in VaD, we hypothesize that TSA may exert its therapeutic effects on VaD by modulating the key pathways of cuproptosis.

Against this backdrop, the present study aimed to investigate the therapeutic effects of TSA on VaD and its association with cuproptosis, with a focus on clarifying whether it inhibits cuproptosis by downregulating the SLC31A1/FDX1 signaling pathway, thereby attenuating neuronal injury and ameliorating cognitive function. This study will provide experimental evidence for uncovering the novel mechanism underlying TSA in the treatment of VaD, and meanwhile offer new strategies and insights for the development of targeted therapy for VaD.

## 2. Materials and methods

### 2.1 Experimental design

**Fig 1:**
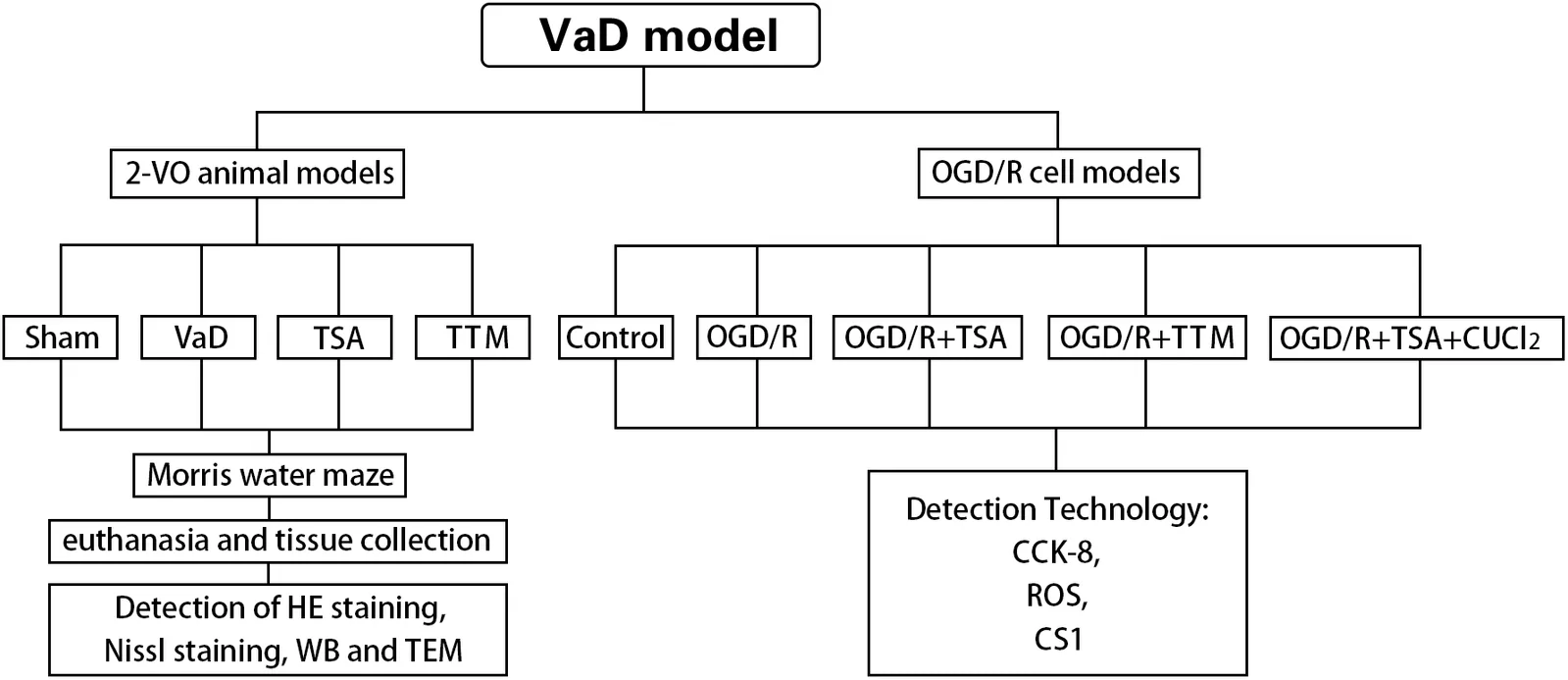
Schematic diagram of the experimental design. (A) In vivo study: rats were divided into sham, VaD, TSA (20 mg/kg), and TTM (10 mg/kg) groups; after 28 days of treatment, Morris water maze, histology (HE/Nissl), TEM, and Western blot were performed. (B) In vitro study: HT-22 cells were subjected to OGD/R with TSA, TTM, or TSA + CuCl_2_ interventions; CCK-8, ROS, and copper accumulation (CS1) were assessed.

The overall experimental design is illustrated in Fig 1.

### 2.2 Chemicals and reagents

TSA (molecular formula: C19H18O3) was purchased from Shandong Sikejie Biotechnology Co., Ltd., with the product batch number: SJ-MN0292 and CAS number: 568-72-9.

**Fig 2:**
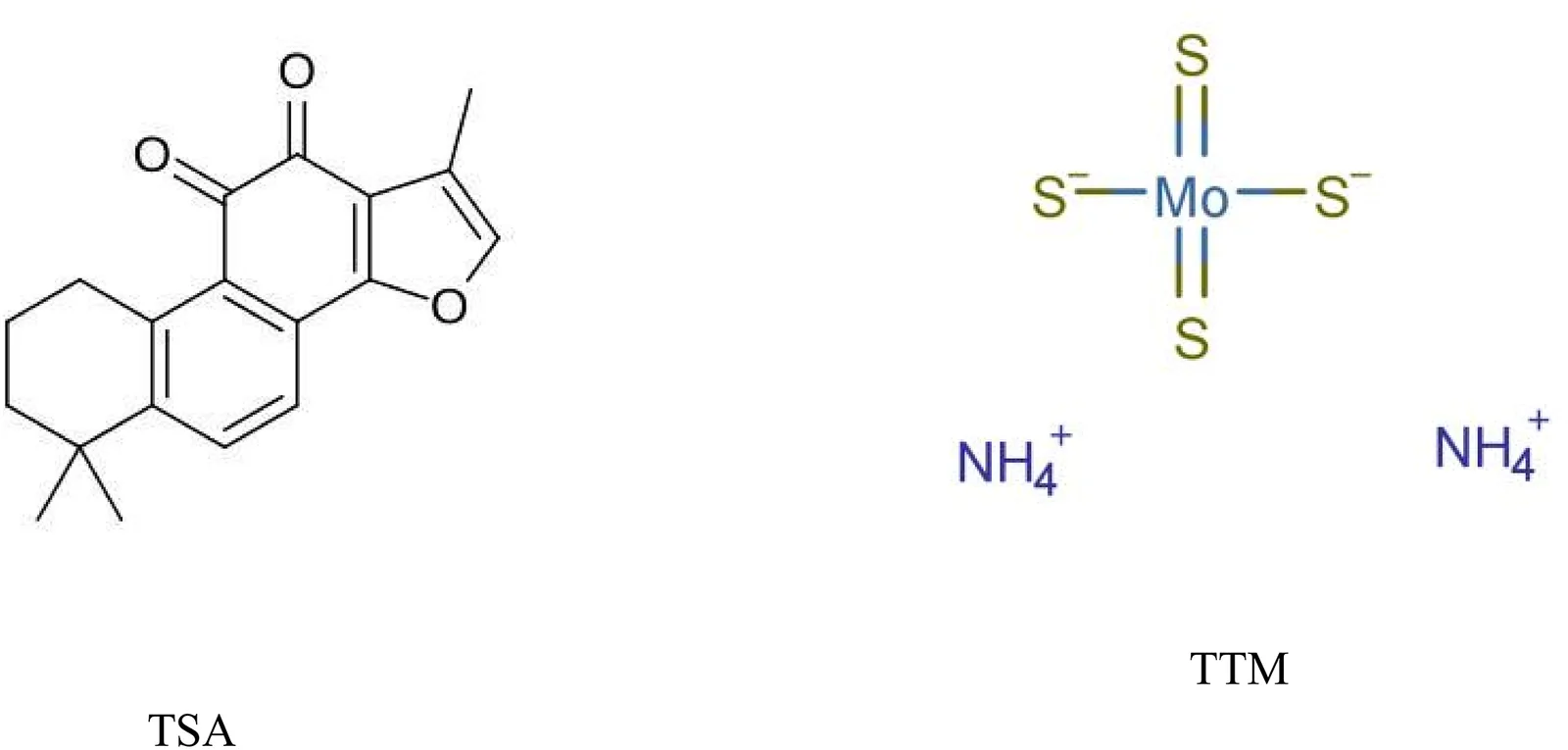
The chemical structures of Tanshinone IIA (TSA) and Tetrathiomolybdate (TTM). (A) Chemical structure of TSA (molecular formula: C_19_H_18_O_3_; molecular weight: 294.34 g/mol). (B) Chemical structure of TTM (molecular formula: (NH_4_)_2_MoS_4_; molecular weight: 244.18 g/mol). TTM was used as a positive cuproptosis inhibitor in this study.

Cuproptosis inhibitor (molecular formula: (NH4)2MoS4) was purchased from Shandong Sikejie Biotechnology Co., Ltd., with the batch number: SJ-MX6196A and CAS number: 15060-55-6 (Fig 2).

### 2.3 Construction of a rat model of VaD

Forty specific pathogen-free (SPF) male Sprague-Dawley (SD) rats, 7 weeks old with a body weight of approximately 250–300 g, were purchased from Henan Skibeth Biotechnology Co., Ltd. (License No.: SCXK (Yu) 2020−0005). The experimental protocol was approved by the Laboratory Animal Welfare and Ethics Committee of Hebei University of Chinese Medicine (Approval No.: DWLL202507006).

A VaD animal model was established via the permanent bilateral common carotid artery ligation (2-VO) method. The detailed procedure was as follows: SD rats were fasted for 12 hours and deprived of water for 4 hours prior to surgery. Anesthesia was induced by intraperitoneal injection of 1% sodium pentobarbital at a dose of 0.4 mL per 100 g body weight. To maintain an adequate surgical plane of anesthesia during the procedure, additional inhaled isoflurane (1–2% in 100% O_2_) was administered via a nose cone as needed. The anesthetized rats were fixed on the operating table in a supine position, with the cervical hair shaved and the skin prepared; routine disinfection was performed with iodophor and alcohol subsequently. A midline incision was made on the neck, and the skin, muscles and connective tissues were bluntly dissected to expose the bilateral common carotid arteries, with the vagus nerves of the rats avoided as much as possible during the procedure. The bilateral common carotid arteries were ligated with 4−0 silk sutures, and the vital signs of the rats were closely monitored thereafter. The cervical muscles and skin were sutured layer by layer, and 2000 units of gentamicin were injected subcutaneously at the wound site for the prevention of wound infection after suture. For the sham group, only the bilateral common carotid arteries were dissected without ligation.

Animal health and behavior were monitored daily (morning and evening) by trained staff. Body weight was measured twice weekly, and neurological function was assessed weekly using the modified Neurological Severity Score (mNSS).

All procedures were performed in accordance with the ARRIVE guidelines. Efforts to minimize suffering included: (1) use of the combined pentobarbital-isoflurane anesthetic protocol as described above; (2) post-operative analgesia with buprenorphine (0.05 mg/kg, s.c., every 12 h for 3 days); (3) ad libitum access to food and water, with soft chow provided for 1 week post-surgery; (4) housing in a temperature- and humidity-controlled environment (22 ± 2°C, 55 ± 5% humidity) with a 12 h light/dark cycle.

All research staff involved in animal care and handling completed a specialized training program on laboratory animal welfare, surgical techniques, and humane endpoint assessment, certified by the Institutional Animal Care and Use Committee (IACUC).

During the experiment, 3 out of 40 rats died before reaching the predefined humane endpoints. Post-mortem examination revealed that 2 deaths were attributed to cerebral hemorrhage secondary to vascular occlusion, and 1 death was due to postoperative infection.

### 2.4 Groups and animal treatments

Forty rats were randomly assigned to four groups (n = 10 per group) using a SPSS-generated random number table, including the sham group,VaD group,TSA group(20 mg/kg), and TTM group(10 mg/kg).The dosage of TTM (10 mg/kg) was selected based on previous studies demonstrating effective copper chelation and neuroprotection at this dose in rat models. Specifically, TTM at 10 mg/kg has been shown to significantly reduce copper accumulation in various tissues and confer neuroprotection in cerebral ischemia models. This dose falls within the range of 1–12 mg/kg previously reported to be well-tolerated in rats without significant systemic toxicity. TTM was administered via intragastric gavage at a dose of 10 mg/kg once daily for 28 consecutive days. All animal modeling was performed via the two-vessel occlusion (2-VO) method. After successful model establishment, rats in the TSA group and TTM group received intragastric administration of corresponding doses of drugs once daily for 28 consecutive days, whereas the sham group and VaD group were given an equal volume of normal saline by gavage. To ensure rigorous experimental blinding, group allocation and drug administration were completed by an independent investigator who was not involved in subsequent outcome assessment. All Morris water maze behavioral tests were video-recorded and analyzed offline by two trained technicians blinded to the experimental grouping. In addition, all histological and WB analyses were performed by operators who remained unaware of the group assignments until the completion of data acquisition.

Humane endpoint criteria for euthanasia: Rats were euthanized if any of the following criteria were met: (1) ≥20% body weight loss from baseline; (2) inability to access food or water for 24 hours; (3) neurological deficit score (mNSS) ≥13 (indicating severe motor/cognitive impairment); (4) persistent hypothermia (body temperature <35°C); or (5) signs of severe distress (e.g., unresponsive to tactile stimulation, labored breathing).

Upon meeting endpoint criteria, rats were immediately transported to the procedure room and euthanized via intraperitoneal injection of pentobarbital sodium (150 mg/kg) within 30 minutes to minimize suffering.

### 2.5 Morris water maze test

The Morris water maze test was conducted after 28 days of continuous administration. The water depth was approximately 35 cm and the water temperature was maintained at around 22°C, with a camera used to track the rats’ positions. The captured images and videos were immediately transmitted to the experiment-specific computer, and data analysis was performed via a water maze analysis system. The water tank was equally divided into four quadrants, with a hidden platform placed in one of them. In the place navigation test, the midpoint of each quadrant was marked as the entry point, and the escape latency—defined as the time taken for the rats to locate the platform and remain on it for more than 2 seconds within 2 minutes—was recorded. This test was performed four times per day, and the average value was calculated. In the spatial probe test, the original platform was removed; the rats were placed into the water from a random entry point, and the number of times they crossed the original platform location and their residence time there within 2 minutes were recorded.

### 2.6 HE staining

Fresh brain tissues randomly selected from each group were fixed in 4% paraformaldehyde solution, then embedded in paraffin and sectioned using a paraffin microtome (PM-24, Servicebio, China) at a thickness of 3 μm. For histological evaluation, five rats per group were randomly selected. The sample size of five animals per group for histological analysis was determined based on commonly accepted standards in rodent histopathological studies, where 5–10 animals per group are generally recommended to ensure reliable and statistically meaningful results. This sample size is consistent with previous studies in the vascular dementia rat model employing similar histological endpoints. Furthermore, to account for potential technical variability and tissue loss during sectioning and staining procedures, three non-adjacent coronal sections per animal and three random high-power fields per section were analyzed, yielding a total of 45 fields per group for quantitative assessment. This multi-level sampling strategy (animal → section → field) enhances the representativeness of the data and compensates for within-animal and inter-section variability, thereby increasing the statistical power of the morphological analyses.From each rat, three non-adjacent coronal sections (200 μm apart) containing the hippocampal formation were collected. Slices were subjected to deparaffinization and dehydration procedures, followed by hematoxylin and eosin staining. After the staining solution was washed, the slices were again dehydrated with gradient alcohols and cleared with xylene before being mounted with resin. All images were captured using a light microscope (SWE-CX63, Servicebio, China). Panoramic views were obtained at ×200 magnification, and high-magnification detailed observations were performed at ×400 magnification. Scale bars (200 μm for ×200 images and 20 μm for ×400 images) were added to all representative micrographs. For quantitative analysis of neuronal damage, the hippocampal CA1 subregion was defined as the region of interest (ROI). Neuronal damage was scored blindly based on the following criteria: 0 = no obvious damage; 1 = mild (cytoplasmic shrinkage, minor nuclear condensation); 2 = moderate (significant pyknosis, irregular cell shape); 3 = severe (extensive karyolysis, cell loss, and inflammatory infiltration). All scoring was performed independently by two investigators blinded to the experimental groups, and the average scores were used for statistical analysis.

### 2.7 Nissl staining

Five rats were randomly selected from each group for Nissl staining.The sample size of five animals per group was chosen to balance statistical robustness with the ethical principle of Reduction (3Rs), and is consistent with published recommendations for histological quantification in rodent models. To maximize the information obtained from each animal and minimize Type II error, three non-adjacent sections per animal and three random high-power fields per section were analyzed, providing a total of 45 quantified fields per experimental group. This hierarchical sampling design increases the effective sample size for statistical comparison and reduces the risk of sampling bias. Rats were anesthetized and fixed in a supine position for transcardial perfusion. After perfusion, the rats were decapitated to harvest the brains, which were then fixed in 4% paraformaldehyde solution. Following paraffin embedding, coronal sections were cut at 5 μm thickness using a microtome (PM-24, Servicebio, China). The sections were subjected to dewaxing and hydration by sequential immersion in xylene, graded ethanol, and distilled water. The sections were stained with methylene blue stain for 10 minutes and differentiated with Nissl differentiation solution until the Nissl bodies were clearly visible under the microscope. Subsequently, the sections were treated with ammonium molybdate solution for 3–5 minutes, followed by routine dehydration, clearing and mounting. Micrographs were captured under a light microscope (SWE-CX63, Servicebio, China) at ×400 magnification, with a scale bar of 20 μm. For quantitative analysis of surviving neurons, the CA1 region of the hippocampus was delineated as the ROI using Image-Pro Plus 6.0 software (Media Cybernetics, USA). Healthy surviving neurons were identified based on the following inclusion criteria: (1) intact and clear cell body contour; (2) visible Nissl bodies in the cytoplasm; (3) well-defined nucleus without pyknosis or karyorrhexis. A semi-automated counting protocol was employed: after manual ROI delineation, the software automatically counted cells meeting the preset threshold. From each of the three selected sections per rat, three random non-overlapping high-power fields (×400) were counted, and the average number of surviving neurons per field was calculated for each animal. All quantitative analyses were performed by two independent researchers who were blinded to the experimental group assignments, and the inter-observer consistency was confirmed (Pearson correlation coefficient > 0.90).

### 2.8 Transmission electron microscopy and mitochondrial morphometric analysis

Hippocampal tissues were dissected into 1 mm³ blocks and immediately fixed in 2.5% glutaraldehyde at 4°C for at least 4 hours, followed by post-fixation in 1% osmium tetroxide for 2 hours. After dehydration through a graded acetone series, the specimens were embedded in Epon resin. Ultrathin sections (60–80 nm) were cut, stained with uranyl acetate and lead citrate, and examined under a transmission electron microscope (TEM, HT7800, Hitachi, Japan). For quantitative morphometric analysis, five randomly selected non-overlapping fields per animal (n = 3 rats per group) were captured at a magnification of ×20,000, with ×40,000 images used for detailed cristae observation. Mitochondrial ultrastructural damage was scored blindly and independently by two trained investigators based on a predetermined 0–3 scale: **0**, normal mitochondria (intact double membranes, well-organized cristae, oval or elongated shape); **1**, mild injury (slight swelling, partial cristae disruption); **2**, moderate injury (obvious swelling, fragmented cristae, mild vacuolization); **3**, severe injury (pronounced swelling, extensively disrupted or absent cristae, prominent vacuolization, and membrane rupture). The average damage score per animal was calculated from the five fields and used for intergroup statistical comparisons. The inter-observer consistency was confirmed (Pearson correlation coefficient > 0.90).

### 2.9 Western blot analysis

After the completion of the Morris water maze behavioral tests in rats, the animals were sacrificed by decapitation, and their brains were harvested. The hippocampal tissues were dissected, and total proteins were extracted therefrom. The extracted proteins were separated by SDS-PAGE gel electrophoresis and then transferred onto PVDF membranes. After blocking with 5% non-fat milk in TBST for 2h at room temperature, the membranes were incubated overnight at 4°C with the following primary antibodies: anti-SLC31A1 (rabbit monoclonal, Cohesion Biosciences, Cat# R27288, dilution 1:1000), anti-FDX1 (rabbit monoclonal, Cohesion Biosciences, Cat# CMA4348, dilution 1:1000), anti-DLAT (rabbit monoclonal, Cohesion Biosciences, Cat# CMA4596, dilution 1:1000), anti-LIAS (rabbit polyclonal, Cohesion Biosciences, Cat# CQA9754, dilution 1:1000), and anti-GAPDH (mouse monoclonal, Proteintech, Cat# 60004–1-Ig, dilution 1:2000). After three washes with TBST, the membranes were incubated with HRP-conjugated goat anti-rabbit IgG (Proteintech, Cat# SA00001−2, dilution 1:5000) and HRP-conjugated goat anti-mouse IgG (Proteintech, Cat# SA00001−1, dilution 1:5000) for 1 h at room temperature. The protein bands were visualized using an enhanced chemiluminescence (ECL) substrate and imaged with a chemiluminescence imaging system..The gray values of each protein band were analyzed using Image-Pro Plus 8.0 software, and the relative expression levels of target proteins were calculated after normalization to GAPDH as the internal reference.

### 2.10 In vitro oxygen-glucose deprivation/reoxygenation (OGD/R) model and experimental treatments

To mechanistically validate the involvement of copper homeostasis in the neuroprotective effects of TSA, in vitro experiments were performed using mouse hippocampal neuronal HT-22 cells (ZQ0476, Shanghai Zhongqiao Xinzhou Biotechnology Co., Ltd., China). Cells were maintained in high-glucose Dulbecco‘s Modified Eagle Medium (DMEM, C11995500BT, Gibco, USA) supplemented with 10% fetal bovine serum (FBS, FSP500, ExCell Bio, China) and 1% penicillin-streptomycin at 37°C in a humidified atmosphere containing 5% CO_2_.

The OGD/R model was established as follows. HT-22 cells were seeded into 96-well plates at a density of 3,500 cells/well for CCK-8 assays, or onto glass coverslips in 24-well plates at 2 × 10⁴ cells/well for fluorescence staining. After overnight culture to allow cell attachment, the culture medium of the OGD/R groups was replaced with glucose-free DMEM (BL1124A, Biosharp, China), and cells were placed in a tri-gas incubator (BB150−2TCS, Thermo Fisher Scientific, USA) saturated with 94% N_2_, 5% CO_2_, and 1% O_2_ at 37°C for 4 hours to induce oxygen-glucose deprivation. Following OGD, the glucose-free medium was replaced with high-glucose DMEM containing 10% FBS, and cells were returned to normoxic conditions (95% air, 5% CO_2_) for 24 hours of reoxygenation. For the normoxic control group, cells were cultured in complete high-glucose DMEM under normal atmospheric conditions throughout the entire experiment.

For drug interventions, cells were randomly divided into five experimental groups: (1) Control (normoxia with vehicle), (2) OGD/R (vehicle only), (3) OGD/R + TSA (10 µM TSA, added 2 hours prior to OGD and maintained throughout reoxygenation), (4) OGD/R + TTM (10 µM TTM, administered under the same regimen as TSA), and (5) OGD/R + TSA + CuCl_2_ (10 µM TSA plus 50 µM CuCl_2_, co-incubated during the entire reoxygenation period).

After the respective treatments, cell viability was evaluated using the SuperKine™ Enhanced Cell Proliferation Assay Kit (CCK-8, BMU106-CN, Abbkine, China). The CCK-8 reagent was diluted 10-fold with fresh culture medium, and 100 µL of the diluted solution was added to each well after removing the old culture medium and gently washing once with PBS. Following incubation at 37°C for 40 minutes, the optical density (OD) was measured at 450 nm using a microplate reader (DNM-9602, Beijing Pulang New Technology Co., Ltd., China). Relative cell viability was calculated as a percentage of the control group.

Intracellular reactive oxygen species (ROS) levels were detected using a Reactive Oxygen Species Assay Kit (CA1410, Beijing Solarbio Science & Technology Co., Ltd., China). After treatment, cells on coverslips were washed twice with PBS and incubated with 10 µmol/L DCFH-DA (diluted 1:1000 in serum-free medium) at 37°C for 20 minutes in the dark. After three washes with serum-free medium to remove extracellular probe, cells were counterstained with Hoechst 33342 (10 µg/mL, B8040, Solarbio) for 5 minutes. Following one additional wash, fluorescence images were captured under a fluorescence microscope (Primostar 3, Zeiss, Germany) at 200 × magnification.

To assess intracellular copper accumulation, cells on coverslips were incubated with the Cu ⁺ -specific fluorescent probe Coppersensor-1 (5 µM, HY-141511, MedChemExpress, USA), diluted from a 5 mM stock solution (prepared in DMSO) in serum-free medium, at 37°C for 20 minutes in the dark. After two washes with PBS, cells were counterstained with Hoechst 33342 (10 µg/mL) for 5 minutes, followed by one additional wash with serum-free medium. Fluorescence images were acquired using a fluorescence microscope (Primostar 3, Zeiss, Germany) at 200 × magnification, with excitation/emission wavelengths of 488/530 nm for Coppersensor-1 and 405/460 nm for Hoechst. Quantitative analysis of fluorescence intensity was performed using ImageJ software (NIH, USA).

All in vitro experiments were performed in triplicate wells and repeated at least three independent times. Statistical analysis was conducted using one-way analysis of variance (one-way ANOVA) followed by Tukey‘s post-hoc test, with significance set at *P* < 0.05.

### 2.11 Statistical analysis

All data were analyzed and graphed using SPSS 23.0 statistical software and GraphPad Prism 8.0. Numerical variables were expressed as the mean ± standard deviation (SD). Prior to parametric analysis, the normality of data distribution was assessed using the Shapiro–Wilk test, and homogeneity of variances was evaluated using Levene’s test. For datasets meeting both assumptions, one-way analysis of variance (one-way ANOVA) followed by Tukey’s post-hoc test was applied for comparisons among multiple groups. For datasets that did not meet the normality assumption, the Kruskal–Wallis H test followed by Dunn’s post-hoc test with Bonferroni correction was used instead. In all figures, significance was set at *P < 0.05 and **P < 0.01 indicate significant differences compared with the VaD group; #P < 0.05 and ##P < 0.01 indicate significant differences compared with the sham group.

### 2.12 Sample size determination

The sample sizes for different experimental modalities were determined based on distinct considerations. For behavioral tests (Morris water maze), all 10 animals per group were included to ensure adequate statistical power for detecting cognitive differences, as previously established in the 2-VO VaD model. For histological analyses (HE and Nissl staining), 5 animals per group were selected, a sample size consistent with published guidelines recommending 5–10 animals per group for immunohistochemical and histomorphometric assessments, and comparable to those used in prior VaD histological studies. For TEM morphometric analysis, 3 animals per group were examined, with 5 randomly selected non-overlapping fields per animal, yielding 15 fields per group for mitochondrial damage scoring—a sampling strategy sufficient to detect significant ultrastructural differences as demonstrated in previous TEM studies. For Western blot analysis, 6 animals per group were used, which falls within the range (3–6 animals per group) commonly employed for protein expression quantification in rodent brain tissues and provides adequate statistical power for detecting meaningful differences in protein levels. All sample sizes were determined prior to the start of the experiment and were not altered during the study.

## 3. Results

### 3.1 TSA alleviated cognitive impairment in VaD rats

“Compared with the sham group, the escape latency in the VaD group was significantly prolonged (##*P* < 0.01), confirming the successful establishment of cognitive impairment. Treatment with TSA or TTM significantly shortened the escape latency compared with the VaD group (*P < 0.05, **P < 0.01), with no significant difference observed between the TSA and TTM groups (*P* > 0.05, Tukey’s post-hoc test).”

**Fig 3:**
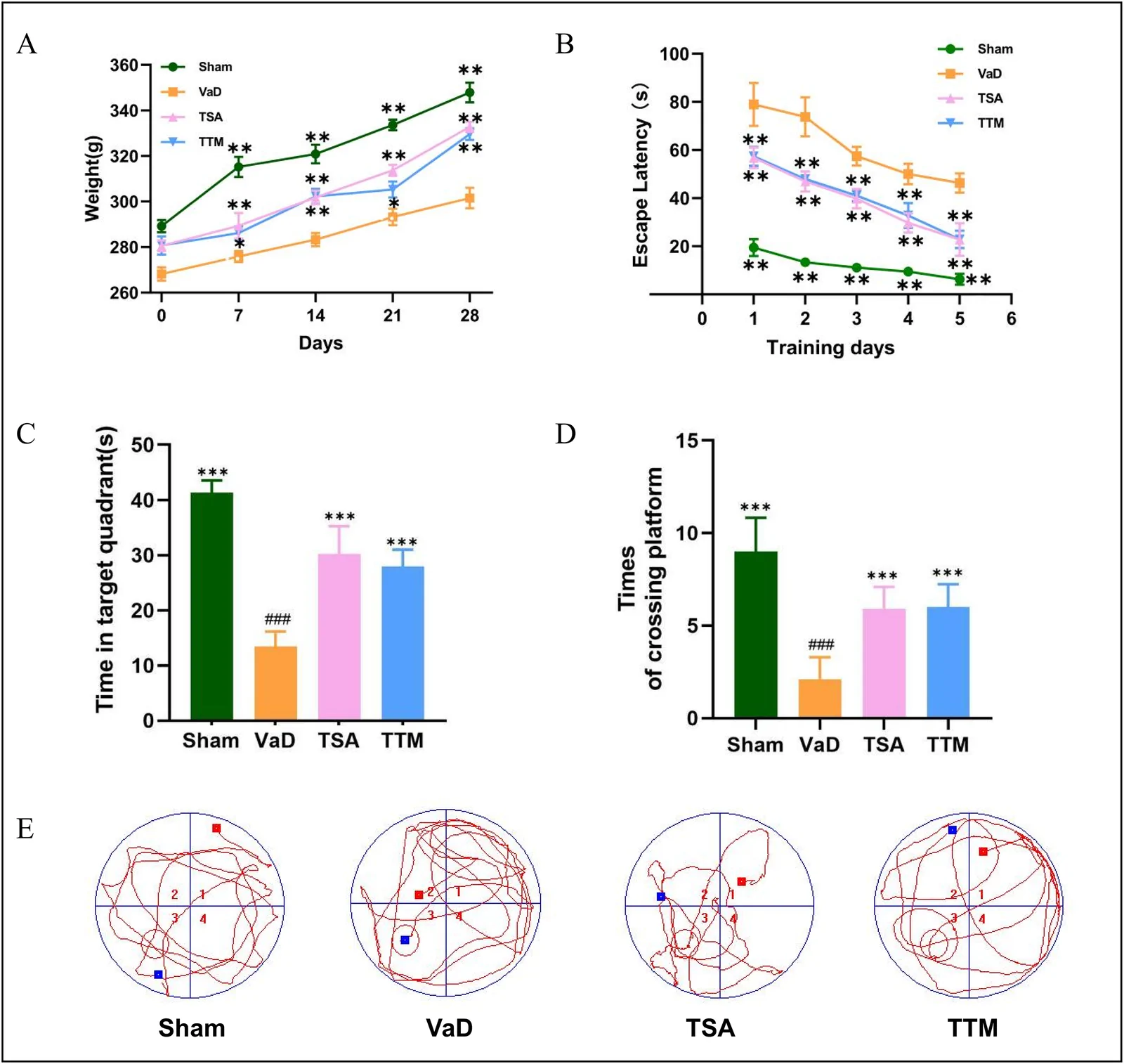
TSA alleviated cognitive impairment in VaD rats. (A) Body weight changes of rats in each group during the 28-day treatment period. (B) Escape latency in the place navigation test over 5 consecutive training days. (C) Percentage of time spent in the target quadrant during the spatial probe test. (D) Number of platform crossings in the spatial probe test. (E) Representative swimming trajectories of rats in each group during the spatial probe test. Data are presented as mean ± SD (n = 10 per group).Statistical significance:*P < 0.05, **P < 0.01,***P < 0.001 vs. VaD group;#P < 0.05, ##P < 0.01, ###P < 0.001 vs.sham group. (one-way ANOVA followed by Tukey’s post-hoc test).

To exclude motor and visual interference on behavioral outcomes, all rats received visual and locomotor screening prior to training; animals with eye lesions or overt movement dysfunction were excluded. A 3-minute free-swimming session without the hidden platform was performed one day before formal testing to alleviate stress responses. Representative swimming trajectories (Fig 3E) revealed that VaD rats swam comparable distances at normal speeds yet displayed disorganized searching routes, confirming their prolonged escape latency stemmed from impaired spatial memory rather than locomotor deficits. Moreover, body weight did not differ significantly between the VaD group and treatment groups, further eliminating poor physical activity as a confounding variable.

Body weight serves as a vital marker of general physical health, and weight dysregulation is strongly correlated with disrupted cerebral homeostasis [19]. We therefore tracked weight fluctuations across all groups. The VaD group exhibited markedly reduced body weight relative to the sham group, whereas TSA and TTM each alleviated this weight loss to varying degrees (Fig 3A), a phenomenon closely tied to disrupted cerebral homeostasis.

We further evaluated the protective effect of TSA against cognitive deficits in VaD rats via the Morris water maze. During place navigation training, VaD rats displayed significantly longer escape latencies versus sham rats, reflecting severe learning impairment. Both TSA and TTM shortened escape latency in a treatment-dependent manner (Fig 3B), verifying their capacity to rescue learning dysfunction. In the spatial probe trial after platform removal, VaD rats spent less time in the target quadrant and crossed the original platform site fewer times than sham animals, consistent with robust deficits in spatial memory for the safe platform location. Representative swimming tracks of each group (Fig 3E) visually illustrate that TSA effectively ameliorates spatial memory damage in VaD rats.

### 3.2 TSA could improve neuronal damage in VaD rats

**Fig 4:**
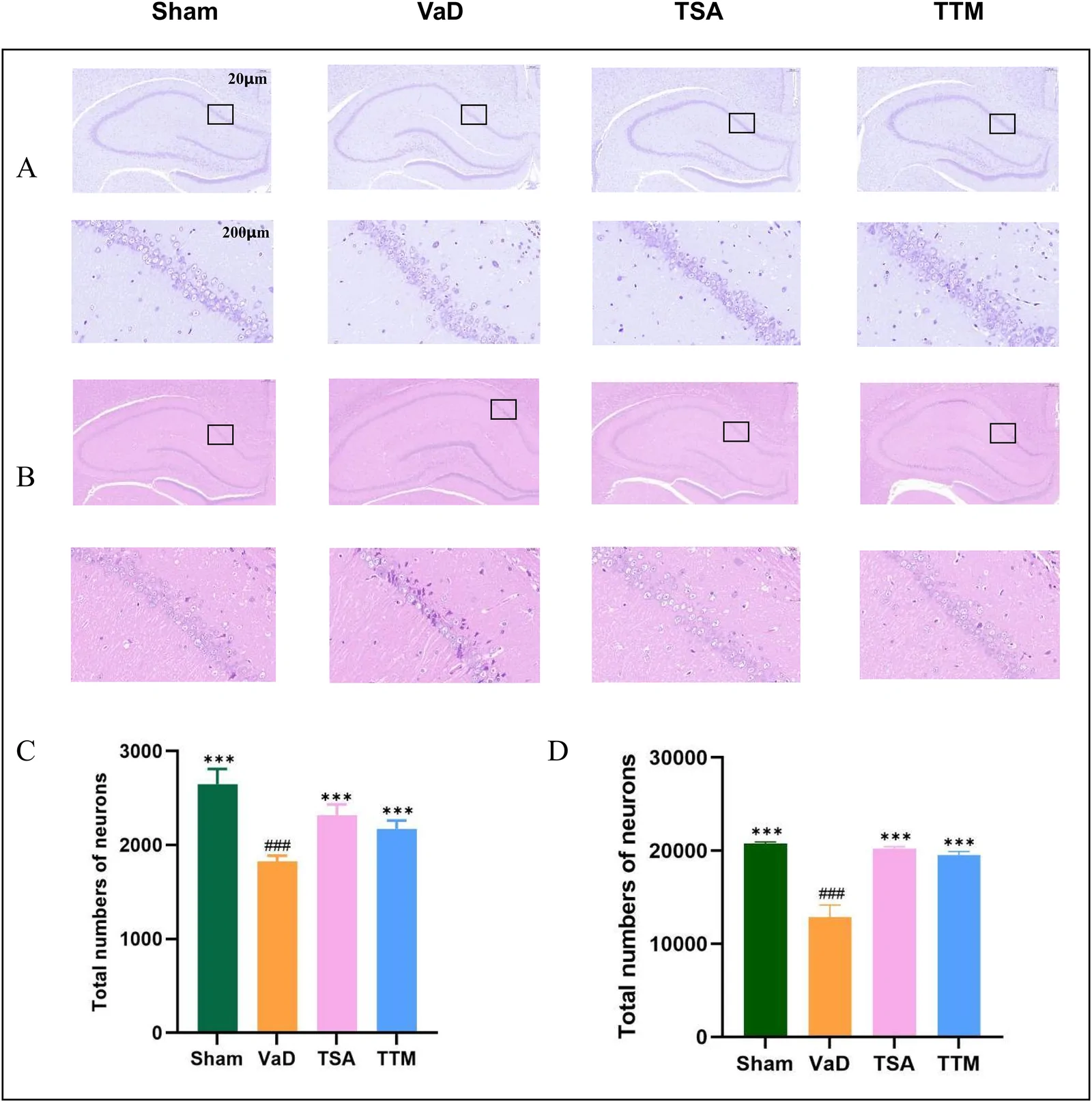
TSA attenuated neuronal damage in the hippocampal CA1 region of VaD rats. Top row: panoramic Nissl staining of rat hippocampus, black box indicates the CA1 observation region, scale bar = 200 μm. Bottom row: magnified Nissl staining images of hippocampal CA1 pyramidal neurons, scale bar = 20 μm. (A) Representative Nissl staining images of hippocampal CA1 neurons. (B) Representative HE staining images of hippocampal CA1 neurons. (C) Quantitative analysis of surviving neuron counts based on Nissl staining. (D) Quantitative analysis of neuronal damage scores based on HE staining. Data are presented as mean ± SD (n = 5 per group).Statistical significance:*P < 0.05, **P < 0.01 vs. VaD group;#P < 0.05, ##P < 0.01 vs. sham group.(one-way ANOVA followed by Tukey’s post-hoc test).

VaD can induce morphological changes and dysfunction of neurons, among which neuronal injury is a key indicator of global cerebral ischemia [20]. In the central nervous system, neurons in the hippocampal CA1 region are particularly sensitive to ischemic and hypoxic stimuli [21]. Therefore, we further investigated the therapeutic effect of TSA on neuronal injury.Nissl staining results showed that, compared with the Sham group, neurons in the CA1 region of the VaD group exhibited hyperchromasia, shrinkage, reduced volume and a significant decrease in number (###*P* < 0.001), indicating severe neuronal injury after 2VO surgery. Treatment with TSA and TTM both significantly reversed the above-mentioned abnormal neuronal changes compared with the VaD group (*P* < 0.05 and *P* < 0.01, respectively; Fig 4A and 4C).Notably, post-hoc analysis revealed that the number of surviving neurons in the TSA group was restored to a level comparable to that of the Sham group, with no statistically significant difference detected between these two groups (*P* > 0.05). Furthermore, no significant difference was observed between the TSA and TTM groups (*P* > 0.05), indicating equivalent neuroprotective efficacy.HE staining revealed that neurons in the Sham group were neatly arranged with plump and intact cell bodies, clear nucleocytoplasmic boundaries, large and round nucleoli with deep and uniform staining, and no inflammatory cell infiltration in the surrounding brain tissue. Compared with the Sham group, the neuronal structure in the CA1 region of the VaD group was severely damaged: the cell bodies were atrophic and irregular in shape, the nucleocytoplasmic boundaries were blurred, nucleolar pyknosis and karyolysis were prominent, a large number of neurons were lost, and extensive inflammatory cell infiltration and interstitial edema were observed in the lesion area, with loose and disordered brain tissue structure (###*P* < 0.001). Treatment with TSA and TTM both markedly reversed the aforementioned abnormal neuronal changes compared with the VaD group (*P* < 0.05; Fig 4B and 4D).Moreover, Tukey’s post-hoc test showed that the neuronal damage scores in the TSA group did not differ significantly from those in the Sham group (*P* > 0.05), nor from those in the TTM group (*P* > 0.05), further confirming the restorative effect of TSA on VaD-induced hippocampal neuronal injury. These findings indicate that TSA can effectively alleviate neuronal injury in rats with VaD and promote an increase in the number of neurons in the hippocampal CA1 region.

### 3.3 Cuproptosis is involved in the therapeutic effect of TSA on VaD rats

Studies have demonstrated that the typical hallmarks of cuproptosis—including mitochondrial morphological and functional alterations, iron-sulfur cluster protein instability, and impaired antioxidant function—are closely associated with oxidative stress injury and the pathophysiological mechanisms of VaD [22]. Among these hallmarks, the SLC31A1/FDX1 signaling pathway is particularly prominent. As a key regulatory pathway of cuproptosis, its abnormal expression level may serve as a crucial pathogenic factor in the onset and progression of VaD. In this study, TEM and WB assays were employed to systematically evaluate the expression changes of cuproptosis-related proteins in the hippocampal tissues of VaD rats, and the interventional effects of TSA and TTM were also investigated.

**Fig 5:**
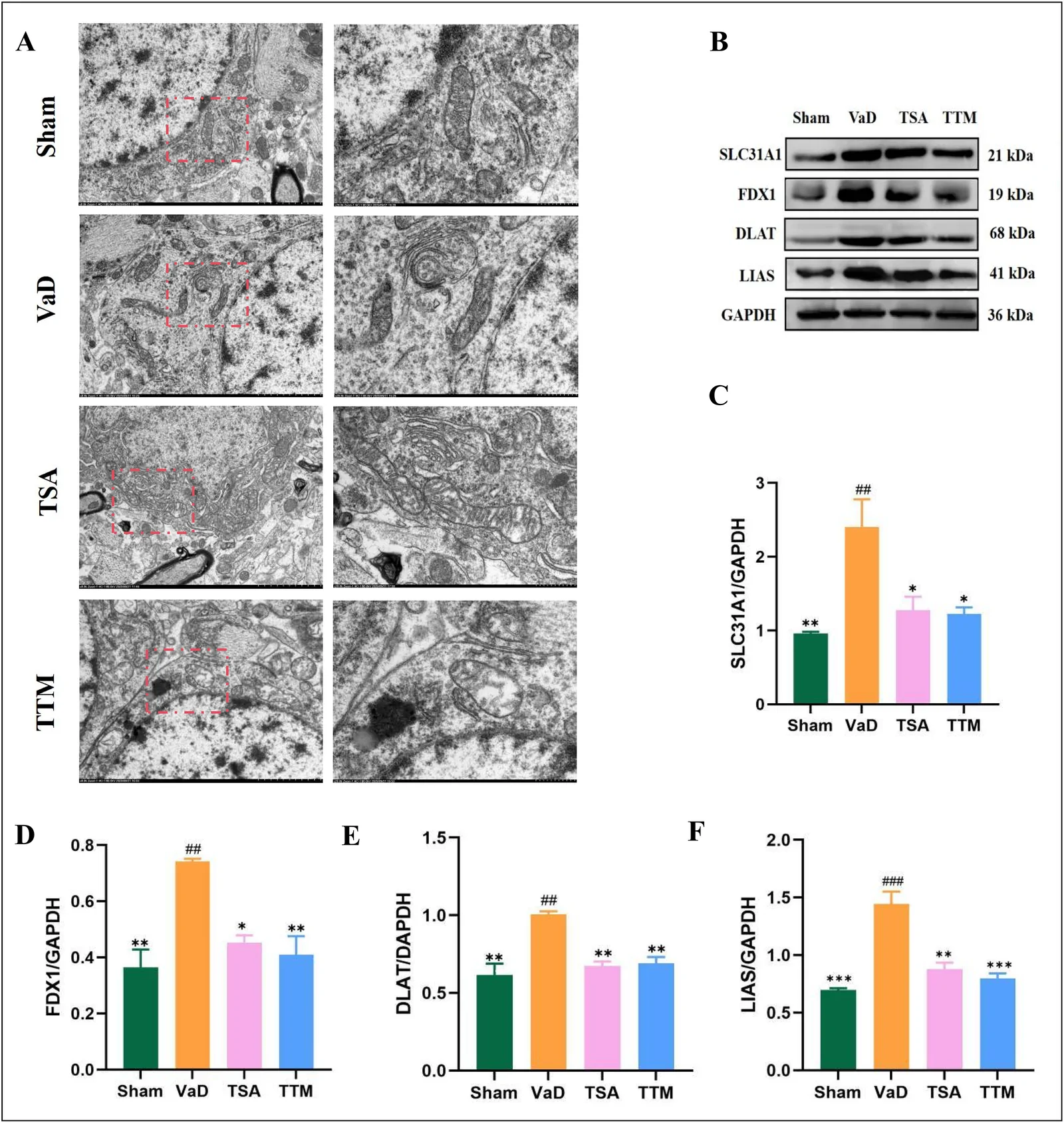
TSA inhibited cuproptosis in hippocampal neurons of VaD rats. (A) Transmission electron microscopy (TEM) micrographs showing mitochondrial ultrastructure in hippocampal neurons of four groups. Left column: low-magnification images, scale bar = 2 μm, red dashed boxes mark the magnified mitochondrial regions; Right column: high-magnification enlarged view of the boxed area, scale bar = 500 nm. Swollen, vacuolated and damaged mitochondria can be observed in the VaD group. Groups from top to bottom: Sham, VaD, TSA, TTM. (B) Representative Western blot bands of cuproptosis-related proteins SLC31A1, FDX1, DLAT and LIAS, with GAPDH as internal loading control. (C–F) Quantitative analysis of relative expression levels of SLC31A1, FDX1, DLAT and LIAS, respectively. All data are presented as mean ± SD, n = 6 per group. Statistical analysis was performed by one-way ANOVA followed by Tukey’s post-hoc pairwise comparisons. *P < 0.05, **P < 0.01, ***P < 0.001 vs. VaD group;#P < 0.05, ##P < 0.01 vs. sham group.

At the ultrastructural level, hippocampal neurons in the VaD group exhibited typical mitochondrial pathological changes compared with the Sham group, characterized by swelling, cristae fragmentation, and vacuolization. Morphometric analysis based on the 0–3 damage scale revealed that the mitochondrial damage score in the VaD group was significantly higher than that in the Sham group (###*P* < 0.001; Fig 5A). After TSA and TTM intervention, the mitochondrial morphology was notably improved, and the quantitative damage scores were significantly reduced compared with the VaD group (*P < 0.05; Fig 5A), suggesting that these two interventions attenuated mitochondrial structural injury, which is one of the ultrastructural features associated with cuproptosis.

At the molecular level, we found that the expression of key cuproptosis regulatory proteins SLC31A1, FDX1, DLAT, and LIAS was significantly upregulated in the VaD group compared with the Sham group (###*P* < 0.01 for SLC31A1 and FDX1; ###*P* < 0.001 for DLAT and LIAS; Fig 5B), confirming excessive activation of the cuproptosis pathway in VaD. Among them, SLC31A1 (CTR1), as a copper ion transporter, showed increased expression, indicating enhanced cellular copper uptake, which provided the material basis for cuproptosis. FDX1 (ferredoxin 1), a core regulator of cuproptosis, is responsible for mediating lipoylation modification. LIAS and DLAT, as key enzymes involved in lipoylation, were upregulated and directly promoted the execution of cuproptosis. These results indicate that excessive activation of the cuproptosis pathway may be one of the important mechanisms underlying neuronal injury in VaD. Notably, both TSA and TTM effectively reversed the abnormal expression of the above proteins compared with the VaD group (*P* < 0.05 or *P < 0.01; Fig 5B). Importantly, Tukey’s post-hoc test revealed no significant differences between the TSA and Sham groups, nor between the TSA and TTM groups, for any of the four proteins measured (all *P* > 0.05), indicating that TSA treatment restored cuproptosis-related protein expression to near-normal physiological levels, comparable to the effect of the positive control TTM. In summary, this study verified the crucial role of cuproptosis in the pathological progression of VaD and revealed that TSA and TTM could reverse the abnormal expression of cuproptosis-related proteins, suggesting their possible involvement in the regulation of this pathway.

### 3.4 TSA reverses the abnormal expression of SLC31A1/FDX1 pathway proteins in VaD rats

TEM-based morphometric analysis further confirmed that the mitochondrial damage score was markedly elevated in the VaD group versus the Sham group (###*P* < 0.001; Fig 5A). This increase was significantly reversed by TSA or TTM intervention (*P < 0.05; Fig 5A), indicating that the protective effects of TSA on mitochondrial ultrastructure were comparable to those of the positive control TTM.

WB results showed that the expression levels of key cuproptosis-related proteins were significantly higher in the VaD group than in the Sham group. The expression of the copper transporter SLC31A1 was markedly increased (###*P* < 0.05), suggesting elevated cellular copper uptake. The expression of the core regulator FDX1 was significantly upregulated (###*P* < 0.05), indicating activation of the cuproptosis pathway. The expression levels of the downstream key lipoylation enzymes LIAS and DLAT were also synchronously and significantly elevated (###*P* < 0.001 and ###*P* < 0.01, respectively), further confirming excessive activation of the cuproptosis pathway. Compared with the VaD group, the overexpression of SLC31A1, FDX1, LIAS, and DLAT was significantly reversed by TSA and TTM intervention (*P < 0.05 or *P < 0.01; Fig 5B–5F), approaching the levels in the sham group. Importantly, Tukey’s post-hoc test further demonstrated that the protein levels of all four markers in the TSA group were restored to values comparable to those in the Sham group, with no significant differences detected between these two groups (all *P* > 0.05). Similarly, no significant differences were observed between the TSA and TTM groups for any of the detected proteins (all *P* > 0.05), indicating that TSA exerts a comparable inhibitory effect on the SLC31A1/FDX1-mediated cuproptotic cascade as the established cuproptosis inhibitor TTM. These results indicate that TSA and TTM can reverse the pathological upregulation of the SLC31A1/FDX1 pathway, which may contribute to the attenuation of copper-induced cellular injury.

### 3.5 Exogenous copper supplementation abolishes TSA-mediated neuroprotection against OGD/R injury in vitro

To functionally validate whether the neuroprotective effects of Tanshinone IIA (TSA) are linked to copper homeostasis regulation, we established an in vitro oxygen-glucose deprivation/reoxygenation (OGD/R) model and challenged TSA-treated cells with exogenous copper (CuCl_2_) supplementation.

**Fig 6:**
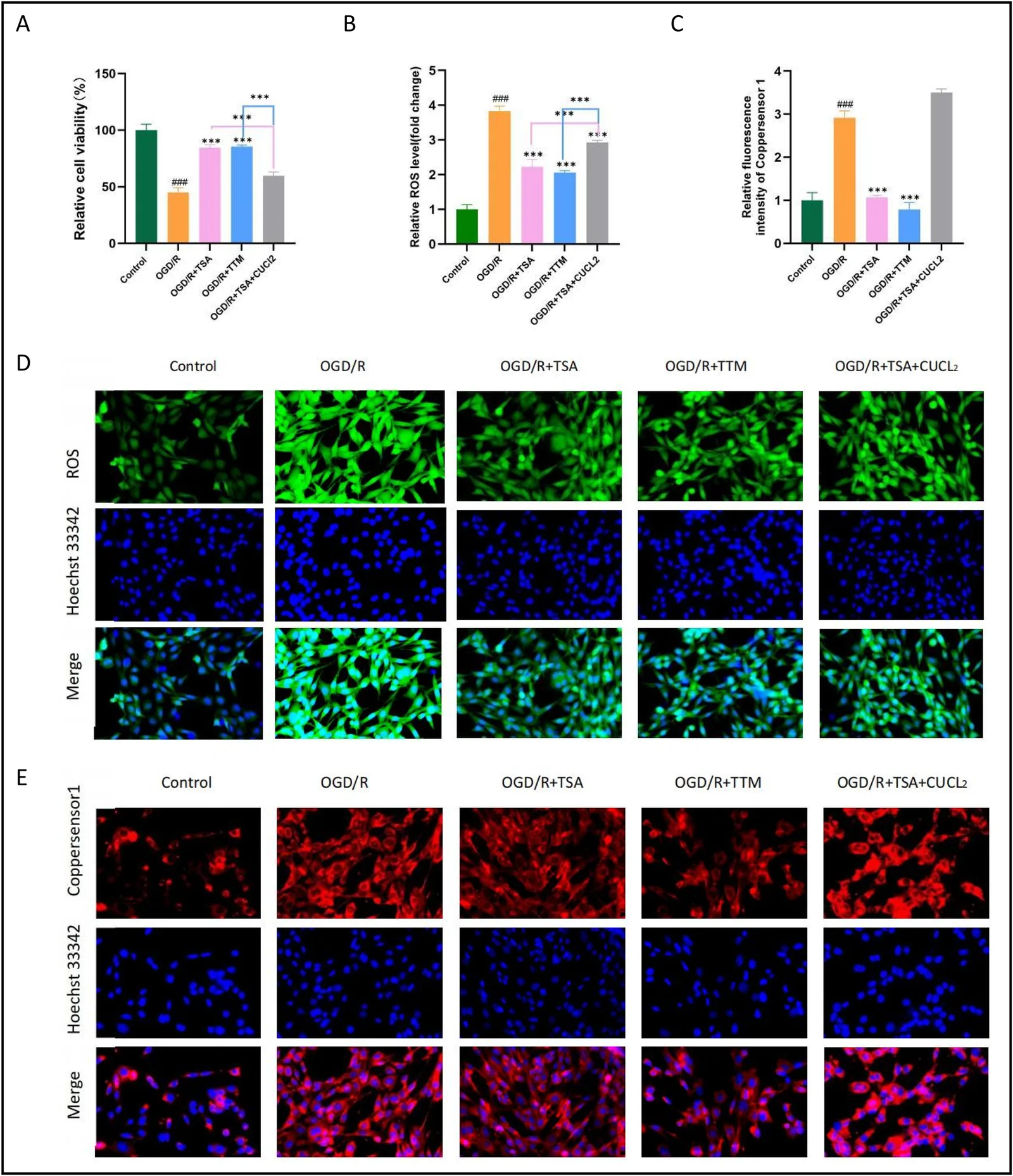
TSA attenuated OGD/R-induced cell injury, oxidative stress, and copper accumulation in vitro. (A-C) Quantitative data for cell viability (CCK-8), ROS levels (DCFH-DA), and Cu⁺levels (Coppersensor-1),respectively.(D,E)Representative fluorescence images of ROS and Coppersensor-1 (green/red) with Hoechst 33342 nuclear staining (blue) in each group (scale bar = XX µm). Data are presented as mean ± SD (n = 3-6). Statistical significance: *P < 0.05, **P < 0.01, ***P < 0.001 vs. OGD/R group; #P < 0.05, ##P < 0.01, ###P < 0.001 vs. Control group (one-way ANOVA with Tukey’s post-hoc test). OGD/R, oxygen-glucose deprivation/reoxygenation; TSA,trichostatin A;ROS, reactive oxygen species.

As presented in (Fig 6A), OGD/R exposure significantly reduced relative cell viability compared with the control group (###*P* < 0.001). TSA treatment markedly reversed this decline, restoring cell survival to near-normal levels. Notably, co-incubation with CuCl_2_substantially abrogated the protective effect of TSA, resulting in a significant decrease in cell viability relative to the TSA-only group (**P < 0.01).

Similarly,OGD/R-induced intracellular reactive oxygen species (ROS) overproduction was effectively suppressed by TSA intervention. However, the addition of CuCl_2_counteracted this antioxidant effect, leading to a robust rebound in ROS levels (Fig 6B). Quantitative analysis of CD11b fluorescence intensity, an indicator of cellular inflammatory stress/activation, exhibited a parallel trend: TSA reduced CD11b expression, whereas CuCl_2_ co-treatment partially reversed this reduction (Fig 6C).

Fluorescence microscopy further corroborated these quantitative findings. As depicted in Fig 6D, intense ROS staining (green) observed in the OGD/R group was markedly attenuated by TSA, but this attenuation was largely nullified upon CuCl_2_ addition. More critically, using a Cu ⁺ -specific fluorescent probe to assess cellular copper(I) deposition (Fig 6E), the OGD/R group displayed pronounced copper accumulation compared to the control group. TSA treatment efficiently reduced this copper signal, suggesting a regulatory effect on intracellular copper retention. However, when exogenous CuCl_2_ was co-administered with TSA, the copper(I) signal was significantly restored, indicating that copper overload directly offsets the protective actions of TSA.

Collectively, these in vitro results demonstrate that the cytoprotective effects of TSA are closely associated with the attenuation of copper-dependent cytotoxicity. The fact that exogenous copper supplementation largely abolishes TSA-mediated neuroprotection provides functional evidence linking TSA activity to copper homeostasis regulation. Nevertheless, these data do not definitively establish engagement of the canonical cuproptosis pathway, but rather support a model in which TSA mitigates copper-induced cellular injury.

## 4. Discussion

VaD the second most prevalent type of dementia worldwide, is characterized pathologically by neuronal loss and brain functional degeneration induced by chronic cerebral hypoperfusion [23]. At present, no specific effective clinical treatment is available. Therefore, further exploration of its pathological mechanisms and the development of targeted therapeutic drugs are of great clinical significance.Abnormal regulation of cell death modes is a key event in the pathological progression of VaD. As a novel form of programmed cell death, cuproptosis is characterized by abnormal copper ion accumulation, mitochondrial dysfunction, and aberrant aggregation of lipoylated proteins. Its association with neurological diseases has been preliminarily confirmed; however, the specific role of the cuproptosis pathway in VaD and strategies for its targeted regulation remain to be elucidated. TSA, the major lipophilic active component of the traditional Chinese medicine Salvia miltiorrhiza, has been proven to alleviate cognitive impairment in VaD rats through anti-inflammatory, antioxidant, and cerebral perfusion-improving effects. The present study, for the first time focusing on the cuproptosis pathway, suggest that TSA may mitigate copper-related cytotoxic stress, as evidenced by the downregulation of SLC31A1/FDX1 pathway proteins and improvement of mitochondrial morphology. However, further direct evidence (e.g., copper content measurement and DLAT lipoylation assay) is required to definitively establish cuproptosis as the primary mechanism. and improving cognitive function in VaD rats. These findings provide a novel explanation for the molecular mechanism underlying TSA in the treatment of VaD and offer new insights for the targeted therapy of VaD.

In the present study, the Morris water maze test first confirmed that TSA significantly improved learning and memory abilities in VaD rats, as evidenced by shortened escape latency, prolonged time spent in the target quadrant, and increased number of platform crossings. Meanwhile, TSA reversed the body weight loss induced by VaD, suggesting that TSA improves the general physiological status and cognitive function of VaD rats at the systemic level. These findings are consistent with the neuroprotective effects of TSA reported in previous studies.Further results from HE staining and Nissl staining showed obvious neuronal atrophy, loss, and inflammatory cell infiltration in the hippocampal CA1 region of VaD rats. TSA intervention markedly restored neuronal number and improved neuronal morphology and structure, verifying that the cognitive protective effect of TSA is closely related to its direct protection of hippocampal neurons. The hippocampal CA1 region is highly sensitive to ischemia and hypoxia and serves as a key brain region responsible for cognitive impairment in VaD. The neuroprotective effect of TSA on this region observed in the present study provides important morphological evidence for its ability to ameliorate cognitive dysfunction in VaD.Mitochondrial dysfunction and disrupted copper ion homeostasis are core hallmarks of cuproptosis. TEM in this study revealed typical mitochondrial swelling, cristae rupture, and vacuolization in hippocampal neurons of VaD rats, whereas TSA intervention significantly restored mitochondrial ultrastructure, indicating that mitochondrial protection is an important mechanism through which TSA exerts neuroprotective effects.At the molecular level, the expression levels of key cuproptosis proteins, including SLC31A1, FDX1, LIAS, and DLAT, were significantly upregulated in the hippocampus of VaD rats, confirming abnormal activation of the cuproptosis pathway in VaD. As a copper transporter, high expression of SLC31A1 promotes excessive intracellular copper accumulation, providing a material basis for cuproptosis. As a core regulator of cuproptosis, FDX1 reduces Cu²⁺to the more toxic Cu⁺and modulates the activity of downstream lipoylation enzymes LIAS and DLAT, thereby promoting aberrant aggregation of lipoylated proteins and instability of iron‑sulfur cluster proteins, ultimately triggering neuronal cuproptosis.Notably, TSA significantly reversed the abnormal overexpression of these proteins, directly demonstrating that TSA inhibits the execution of cuproptosis by suppressing excessive activation of the SLC31A1/FDX1 signaling pathway. This represents a novel mechanism underlying the therapeutic effect of TSA against VaD identified in the present study.

In this study, the copper ion chelator TTM was used as a positive control, and both the selection basis and experimental results are of important scientific significance. As a copper ion chelator, TTM can reduce intracellular copper availability. While TTM is often used in cuproptosis research, it is not a specific pathway inhibitor and may have off-target effects. In this study, TTM served as a positive control for copper chelation rather than as definitive proof of cuproptosis pathway blockade. and block the cuproptosis pathway at the source. In the present study, TTM also improved cognitive function, alleviated neuronal injury, and downregulated the expression of SLC31A1/FDX1 pathway proteins in VaD rats. Its intervention effect was consistent with that of TSA, which not only verified the reliability of the cuproptosis pathway as a therapeutic target for VaD, but also provided an important reference for the mechanism of TSA.Comparing their mechanisms of action, TTM exerts a specific inhibitory effect on cuproptosis only by chelating copper ions, whereas TSA, as a natural active ingredient of traditional Chinese medicine, possesses multiple pharmacological activities including anti‑inflammatory, antioxidant, mitochondrial protective, and cuproptosis‑regulating effects. This multi-pathway mode of action endows it with more significant advantages in the treatment of VaD, especially providing a safer therapeutic option for patients intolerant to chemical inhibitors.The innovation of this study lies in the first correlation between the neuroprotective effect of TSA and the cuproptosis pathway, confirming that the SLC31A1/FDX1 signaling pathway is a key target of TSA in the treatment of VaD. This study supplements the experimental evidence that cuproptosis participates in the pathological progression of VaD, and also enriches the research on the molecular mechanisms by which active ingredients of traditional Chinese medicine regulate novel types of programmed cell death.

The choice of TTM at 10 mg/kg as the positive control was substantiated by multiple independent studies demonstrating its efficacy at this dose in rat models. Notably, this dosage has been validated in the context of cerebral ischemia, where intravenous administration of 10 mg/kg TTM conferred significant neuroprotection and reduced infarct size. However, several considerations regarding TTM’s pharmacological profile warrant discussion. First, TTM acts through a dual mechanism: when administered with food, it forms complexes with copper and dietary proteins, preventing intestinal copper absorption; when given between meals, it is absorbed and directly chelates copper from tissue stores. In the present study, TTM was administered by gavage, which may have partially affected copper absorption from the gastrointestinal tract. Second, while TTM is considered a relatively specific copper chelator, potential off-target effects have been documented, including reversible elevation of transaminases, anemia, and neutropenia. Although we did not observe overt signs of systemic toxicity at the dose used in this study, we cannot completely exclude the possibility of mild, subclinical off-target effects that might have contributed to the observed neuroprotective outcomes. Third, copper is an essential cofactor for multiple physiological processes, including mitochondrial respiration, antioxidant defense, and neurotransmitter synthesis [24]; therefore, chronic copper depletion by TTM could theoretically interfere with these normal functions. Despite these considerations, the comparable efficacy between TSA and TTM in our experimental paradigm supports the involvement of copper homeostasis in the neuroprotective effects observed, without implying that TSA acts through an identical mechanism.

However, this study still has several limitations. First, the present study was only conducted in a rat model of VaD, and the expression changes of the SLC31A1/FDX1 pathway and the intervention effect of TSA have not been verified in clinical samples; therefore, its clinical relevance needs to be further confirmed. Second, this study only clarified the overall regulatory effect of TSA on the SLC31A1/FDX1 pathway, but the specific molecular targets through which TSA regulates this pathway remain to be further explored, such as whether it affects the transcriptional expression of pathway genes by regulating histone acetylation, transcription factor binding, and other processes. Third, this study did not further investigate the synergistic relationship between the cuproptosis-inhibitory effect of TSA and its anti-inflammatory and antioxidant effects, and the interactive regulatory mechanisms underlying its multiple pharmacological activities remain to be elucidated.Based on the results and limitations of this study, future research can be carried out in the following directions. First, expand the sample size of clinical subjects, detect the expression levels of SLC31A1/FDX1 pathway proteins in brain tissues and peripheral blood of VaD patients, and clarify their correlation with clinicopathological characteristics and cognitive function scores of VaD, so as to provide evidence for the cuproptosis pathway as a target for clinical diagnosis and treatment of VaD. Second, further explore the specific molecular mechanism by which TSA regulates the SLC31A1/FDX1 pathway and identify its direct functional targets through experiments such as chromatin immunoprecipitation and dual-luciferase reporter assays. Third, further verify the key role of this pathway in the neuroprotective effect of TSA by establishing VaD animal models with specific knockout or overexpression of SLC31A1 or FDX1. Fourth, explore the combined therapeutic effect of TSA with other cuproptosis inhibitors or VaD therapeutic drugs, so as to provide experimental support for the development of more effective combination regimens for VaD. In addition, the crosstalk between the cuproptosis pathway and other pathological mechanisms in VaD (such as neuroinflammation, oxidative stress, and autophagy) can be further investigated to construct a more comprehensive network of pathological mechanisms underlying VaD.

In summary, this study confirms that abnormal activation of the SLC31A1/FDX1-mediated cuproptosis pathway is an important mechanism underlying neuronal injury and cognitive dysfunction in VaD,and TSA can downregulate the excessive activation of this pathway, reduce neuronal copper overload, and repair mitochondrial dysfunction, thereby inhibiting neuronal cuproptosis, alleviating hippocampal neuronal damage, and ultimately improving cognitive impairment in VaD rats.This study reveals for the first time the cuproptosis-regulating mechanism of TSA in the treatment of VaD. It provides a novel molecular theoretical basis for the application of the traditional Chinese medicine Salvia miltiorrhiza in VaD therapy, develops new potential targets for the targeted treatment of VaD, and offers new insights for research on the regulation of novel types of programmed cell death by active ingredients from natural traditional Chinese medicines.

## 5. Discussion-Limitations

This study has several limitations. First, neither the in vivo nor in vitro experiments included a drug-treated control group (sham operation/normal control plus drug administration). The absence of this group prevents us from completely ruling out the possibility that TSA and TTA exert non-specific baseline pharmacological effects under non-pathological conditions, and impairs our capacity to draw causal inferences that the observed effects represent strict disease-specific reversal.Although our in vitro data (Figures A and B) demonstrate that these compounds, whether used alone or combined with CUCl_2_, only restore cell viability and ROS levels after OGD/R injury to the normal baseline without surpassing it—an indirect indication that their activity may depend on a pathological microenvironment—such indirect evidence cannot substitute for direct experimental controls.Accordingly, we have conservatively framed our conclusions to state that these compounds exert prominent protective effects under OGD/R or ischemic stress, rather than making absolute claims of disease-specific reversal. Future research should incorporate standardized sham-operated drug treatment control groups to further verify the target specificity and pharmacological action profile of these compounds.

Second, only Morris water maze was applied to evaluate cognitive function in this study, which can only reflect spatial learning and memory, and cannot comprehensively evaluate working memory, attention and other cognitive domains affected by vascular dementia. In addition, without additional open field test, Y-maze and novel object recognition test, we cannot quantitatively separate the interference of locomotor activity and emotional stress on behavioral indicators. Further multi-dimensional behavioral tests are required in subsequent research: open field test to evaluate autonomous movement, Y-maze and novel object recognition test to detect working memory and recognition function, so as to more accurately distinguish cognitive deficits from exercise-related confounding factors.

Third, while our TEM morphometric analysis provided semi-quantitative evidence of mitochondrial structural protection by TSA, we did not perform complementary functional assays, such as mitochondrial reactive oxygen species (MitoSOX) detection or mitochondrial DNA (mtDNA) copy number/integrity assessment, which are critical for defining mitochondrial functional status. Therefore, the causal relationship between TSA-mediated mitochondrial structural preservation and cuproptosis inhibition requires further functional validation. Subsequent investigations will incorporate these assays, along with measurements of mitochondrial membrane potential and ATP levels, to comprehensively evaluate mitochondrial function in response to TSA treatment.

Fourth, while the sample sizes for histological analyses (n = 5 per group) are consistent with published standards and sufficient to detect the observed statistically significant differences between groups, we acknowledge that larger sample sizes would further enhance the statistical power and generalizability of the morphometric findings. The relatively small sample size for TEM analysis (n = 3 per group) is a particular limitation, as mitochondrial ultrastructural assessment is inherently more variable and susceptible to sampling bias. Although our hierarchical sampling strategy (5 fields per animal, 3 animals per group) partially mitigates this concern, future studies with larger TEM sample sizes and complementary functional assays (e.g., MitoSOX, mtDNA copy number) would strengthen the conclusions regarding mitochondrial protection.

The consistent changes of cognitive behavior, hippocampal neuronal morphology, mitochondrial ultrastructure and cuproptosis-related molecular proteins jointly confirmed that the intergroup differences in Morris water maze originated from neuronal injury-induced cognitive dysfunction, rather than the interference of movement, vision or stress.

Fifth, and most importantly, the mechanistic evidence for cuproptosis in this study remains indirect. According to the established cuproptosis criteria, direct evidence requires: (1) measurement of copper content in hippocampal tissues (e.g., ICP-MS) to confirm copper overload; and (2) detection of DLAT lipoylation aggregation, which is the hallmark event of cuproptosis. However, due to the limited availability of hippocampal tissue samples and the prolonged procurement time for specific antibodies against lipoylated DLAT, we were unable to complete these assays in the current study. Therefore, although our results demonstrate that TSA modulates cuproptosis-related protein expression (SLC31A1/FDX1) and improves mitochondrial ultrastructure, we cannot definitively conclude that TSA exerts its effects exclusively via the canonical cuproptosis pathway. Instead, we conservatively interpret our findings as indicating that TSA alleviates copper-dependent cytotoxicity and restores copper homeostasis. We have accordingly revised the language throughout the manuscript to reflect this limitation, toning down causal claims and emphasizing that the precise molecular mechanism warrants further investigation.

## Supporting information

S1 File Supplementary raw data. This file contains the raw data underlying the results presented in this study, including: CCK-8 cell viability assay data (CCK8.xls), copper content in hippocampal tissues (Copper content values.xlsx), Morris water maze behavioral test data (Morris water maze data.xlsx), quantification of Nissl-stained surviving neurons (Quantification of Nissl-stained neurons.xlsx), quantitative analysis of neuronal damage scores (Quantitative analysis of neuronal damage scores.xlsx), rat body weight records during the treatment period (Rat body weight.xlsx), and reactive oxygen species (ROS) fluorescence intensity data (ROS.xlsx). All data are provided in the compressed Supplementary Materials.zip file.
(ZIP)
